# Supplementary figures and images for: Anti-Inflammatory Effects of Allocryptopine via the Target on the CX3CL1–CX3CR1 axis/GNB5/AKT/NF-κB/Apoptosis in Dextran Sulfate-Induced Mice
Source: Biomedicines. 2023 Feb 5;11(2):464. doi: 10.3390/biomedicines11020464 (PMC9952939; doi:10.3390/biomedicines11020464)

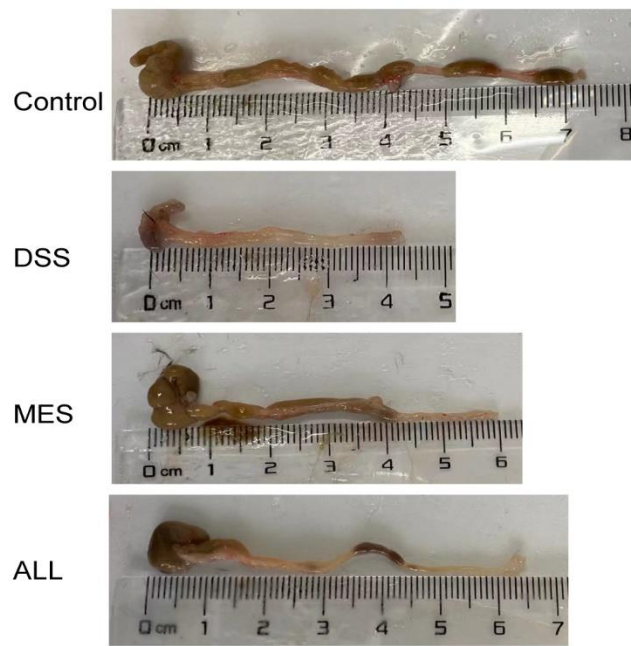

**Supplementary Figure S1.** ALL and MES improved the colon length of DSS-induced colitis in C57BL/6 mice.

Supplement: Supplementary file 1 [file biomedicines-11-00464-s001.zip › biomedicines-2154661-supplementary Figures.pdf]
